# Pulmonary sarcomatoid carcinoma with METex14 mutation and high PD-L1: A case report

**DOI:** 10.1097/MD.0000000000051532

**Published:** 2026-11-06

**Authors:** Junzhang Qin, Zhen Xiong

**Affiliations:** a The Department of Respiratory and Critical Care Medicine, People’s Liberation Army Joint Logistic Support Force 924th Hospital: 181st Hospital of Chinese People’s Liberation Army, Guilin, Guangxi, China.

**Keywords:** case reports, high PD-L1 expression, immunotherapy, MET exon 14 skipping mutation, pulmonary sarcomatoid carcinoma, surgical treatment, targeted therapy

## Abstract

**Rationale:**

Pulmonary sarcomatoid carcinoma (PSC) is a rare and aggressive subtype of lung cancer, posing diagnostic and therapeutic challenges. Cases of PSC harboring both MET exon 14 skipping (METex14) mutation and high PD-L1 expression are rarely documented, and reports detailing multimodal treatment involving surgery, chemotherapy, targeted therapy, and immunotherapy in such patients are even scarcer.

**Patient concerns:**

A 54-year-old man presented with a persistent cough for over half a month. He was previously healthy. Physical examination revealed diminished breath sounds in the right upper lung.

**Diagnoses:**

Initial bronchoscopic biopsy suggested inflammation, and computed tomography (CT)-guided lung biopsy showed atypical giant cells but was inconclusive. Following multidisciplinary team discussion, the patient underwent surgical resection. Postoperative pathology confirmed pleomorphic carcinoma (sarcomatoid carcinoma). Subsequent genetic testing identified a METex14 mutation with high PD-L1 expression (Tumor Proportion Score ≥ 50%). The final diagnosis was stage IIIB (T4N2M0) right PSC, MET-positive, PD-L1-positive.

**Interventions:**

The initial treatment plan included 1 cycle of toripalimab (an anti-PD-1 antibody) combined with docetaxel and lobaplatin chemotherapy. This was subsequently adjusted to boretinib (a MET inhibitor) targeted therapy combined with docetaxel and lobaplatin chemotherapy.

**Outcomes:**

The patient achieved and maintained a partial response (PR) for over 6 months of follow-up. Symptoms including cough resolved, and his general condition remained good with satisfactory appetite and sleep.

**Lessons:**

This case underscores the diagnostic difficulty of PSC, which is prone to misdiagnosis and exhibits rapid growth. Surgery may not be appropriate for unresectable stage IIIB PSC. Gene sequencing and PD-L1 testing are crucial as they expand therapeutic options for PSC patients. The optimal first-line therapy (targeted vs immunotherapy) for PSC with concurrent MET mutation and high PD-L1 expression remains undefined, warranting further exploration of combination strategies.

## 1. Introduction

Pulmonary sarcomatoid carcinoma (PSC) is a rare subtype of non-small cell lung cancer (NSCLC), characterized by poor differentiation, aggressive progression, and an incidence of <1%.^[1]^ It poses significant challenges in both diagnosis and treatment, with a poor prognosis. Cases of PSC harboring concurrent MET exon 14 skipping mutation and high programmed death-ligand 1 (PD-L1) expression have been rarely documented. Even fewer reports describe patients with such dual-positive PSC who have undergone multimodal therapy involving surgery, chemotherapy, targeted agents, and immunotherapy. In this context, we conducted a retrospective analysis of the clinical data from a patient admitted to the 924th Hospital of the Joint Logistics Support Force of the Chinese People’s Liberation Army. This report, supplemented by a review of relevant literature, aims to provide insights and references for clinical practice.

## 2. Case report

A 54-year-old man presented with a persistent dry cough for over half a month. He reported the onset of the cough, initially nonproductive and later producing scant white mucus, in early July 2025. A chest CT performed at a local hospital revealed a “space-occupying lesion in the right upper lobe” of the lung. His past medical history was unremarkable. Physical examination on admission revealed stable vital signs and normal mental status. Diminished breath sounds were noted in the right upper lung field, with no rales audible in either lung. Cardiac and abdominal examinations were normal, and there was no edema in the lower extremities. Routine blood tests showed a white blood cell count of 9.14 × 10^9^/L, hemoglobin of 118 g/L, platelet count of 290 × 10^9^/L, and neutrophil count of 7.17 × 10^9^/L. Laboratory findings included an elevated C-reactive protein (CRP) level of 39.21 mg/L, hypoalbuminemia (33.9 g/L), and mild hypokalemia (3.28 mmol/L). The serum tumor-specific growth factor was elevated at 79.33 U/mL, while other tumor markers, renal function tests, cardiac enzymes, coagulation profile, and routine urinalysis and stool tests were within normal limits. No pathogens were isolated from sputum or bronchoalveolar lavage fluid. An electrocardiogram showed normal sinus rhythm. Echocardiography revealed no abnormalities in cardiac chamber sizes. Abdominal ultrasound identified a solid hepatic nodule suggestive of hemangioma. The pancreas, spleen, kidneys, and ureters were unremarkable, and no significant lymphadenopathy was detected in the bilateral cervical, supraclavicular, or retroperitoneal regions. Chest CT demonstrated a 6.8 cm × 9.4 cm, well-circumscribed, lobulated soft-tissue mass with heterogeneous density and surrounding ground-glass opacity in the right upper lobe, accompanied by ipsilateral mediastinal lymphadenopathy (Fig. 1A, C, D). Contrast-enhanced abdominal CT suggested a possible hemangioma in the left hepatic lobe and a right renal mass suspicious for clear cell carcinoma. Head CT showed no significant abnormalities. An endobronchial ultrasound-guided biopsy of the right upper lobe lesion was performed on July 23, 2025, demonstrating inflammatory changes (Fig. 2). A subsequent CT-guided biopsy of the same lesion on August 6 revealed significant interval growth of the mass to 9.9 cm × 8.1 cm (Fig. 1B). The limited biopsy specimen, supported by immunohistochemistry, raised suspicion for sarcomatoid carcinoma (Fig. 3). The tumor cells were positive for CK and Vimentin, and negative for EMA, CK5/6, CK7, TTF-1, NapsinA, ALK, P40, CD56, Syn, and CgA. INI1 and BRG-1 were retained. The Ki-67 proliferation index was approximately 50%. Alcian blue-periodic acid-Schiff staining was negative. Comprehensive tumor genomic profiling and PD-L1 testing conducted on August 7, 2025, identified a MET exon 14 skipping mutation (c.3028 + 1G>T, variant allele frequency 27.2%) and high PD-L1 expression with a Tumor Proportion Score (TPS) of 50%. Following the procedure, the patient developed pneumothorax with pleural effusion, which was managed with closed thoracic drainage. A multidisciplinary team (MDT) discussion was subsequently convened. The team concluded that: the diagnosis of malignant lung tumor was definitive, classified as stage IIIB; given the large size of the pulmonary mass and the high suspicion for PSC based on prior biopsies, a definitive histologic subtype was not yet confirmed; and surgical resection was indicated per the Chinese Society of Clinical Oncology (CSCO) guidelines. Consequently, a right upper lobectomy with lymph node dissection was performed on August 12, 2025.

**Figure 1.**
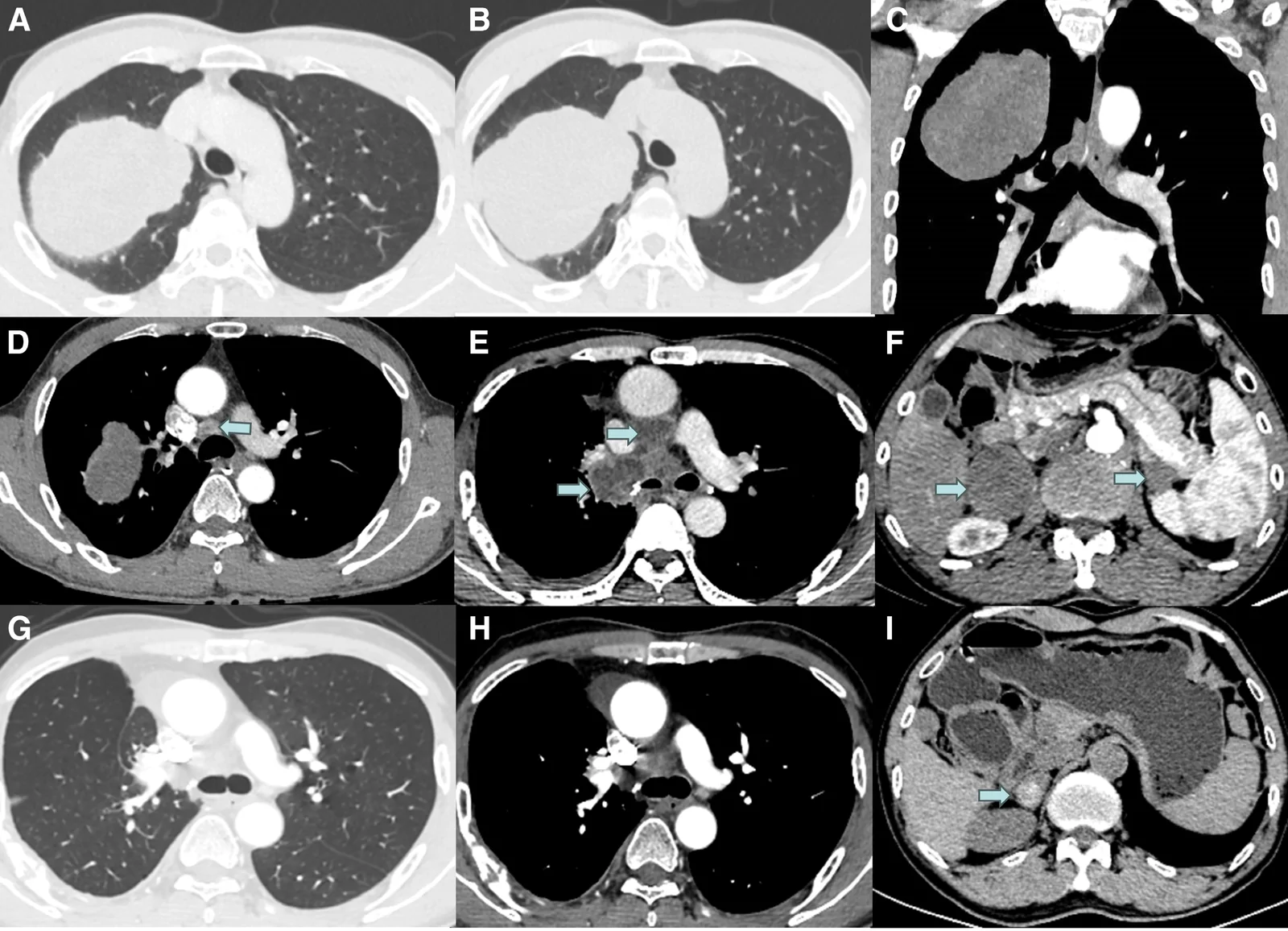
Serial chest and abdominal CT findings in a patient with PSC. (A) Initial CT scan (July 22, 2025) shows a right upper lobe mass measuring 6.8 cm × 9.4 cm with surrounding ground-glass opacity. (B) Rapid tumor growth (9.9 cm × 8.1 cm) is noted on August 6, 2025. (C) Coronal view clearly depicts the anatomical relationship of the right upper lobe mass to surrounding structures. (D) Ipsilateral mediastinal lymphadenopathy is present. (E) Follow-up CT scan (September 22, 2025) reveals enlarged, confluent mediastinal lymph nodes (largest 6.2 cm × 5.1 cm) causing tracheal compression. (F) Bilateral adrenal metastases are identified, the largest measuring 5.1 cm × 4.2 cm. (G) Post‑lobectomy changes with compensatory expansion of the right middle lobe (December 26, 2025). (H) Significant reduction in mediastinal lymph node size after treatment. (I) Marked shrinkage of bilateral adrenal metastases,with the largest measuring 2.2 cm × 1.8 cm. CT = computed tomography, PSC = pulmonary sarcomatoid carcinoma.

**Figure 2.**
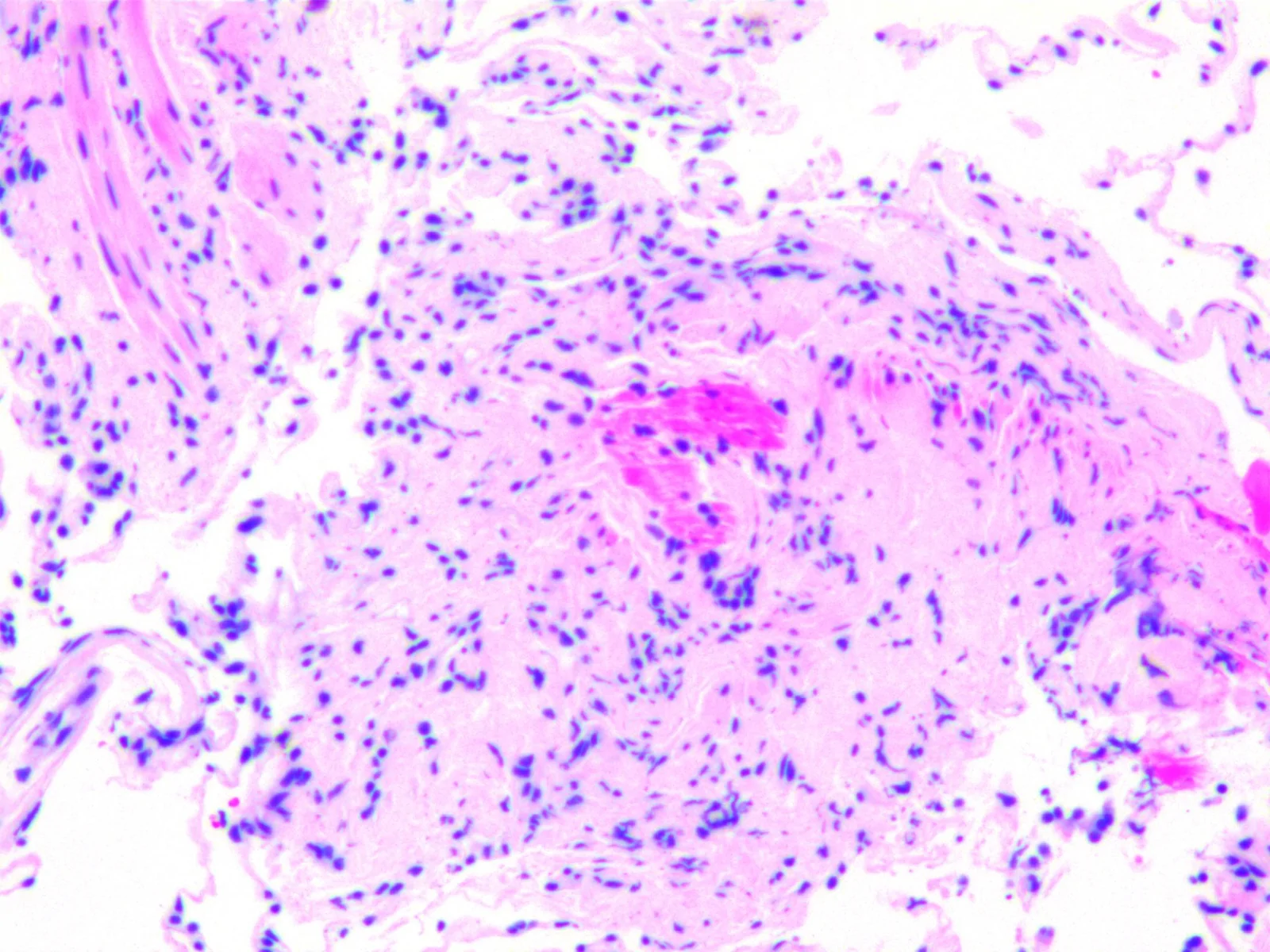
Histopathological examination (hematoxylin and eosin stain) of the initial bronchoscopic biopsy specimen. The image shows widened alveolar septa with focal fibrosis. The interstitium is infiltrated by scattered lymphocytes and histiocytes. Anthracotic pigment deposition is also noted. (original magnification, ×200).

**Figure 3.**
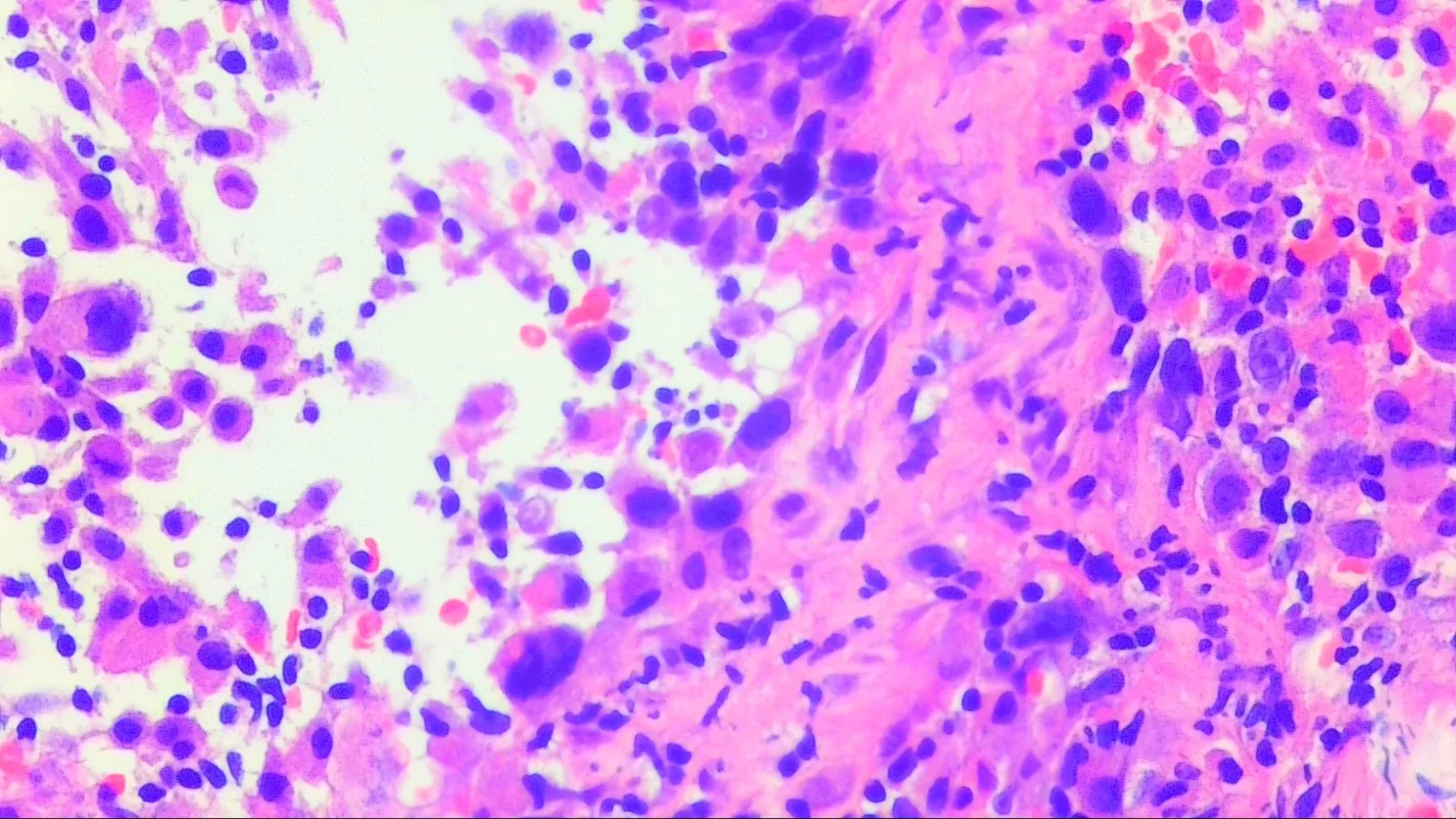
Histopathological findings of the CT-guided lung biopsy specimen (hematoxylin and eosin stain). The image shows residual normal alveolar walls alongside areas of patchy necrosis, accompanied by infiltration of numerous neutrophils and lymphocytes. Scattered among these are highly atypical tumor cells characterized by large cellular volume, abundant cytoplasm, and pleomorphic morphology. Mitotic figures are present, and tumor giant cells are also evident. (original magnification, ×200). CT = computed tomography.

Intraoperatively, fusion of the right upper lobe bronchus and pulmonary vein was observed, resulting in obscured anatomy that complicated complete dissection. The gross specimen revealed extensive necrotic tissue with cystic degeneration and a spongy, paste-like consistency on sectioning (Fig. 4). Postoperative histopathology confirmed a poorly differentiated carcinoma. Combined with immunohistochemical findings, the diagnosis was consistent with pleomorphic carcinoma (sarcomatoid carcinoma). Metastatic carcinoma was identified in lymph node stations 2/4, 7, and 10 (Fig. 5). The final diagnosis was Right PSC, Stage IIIB (T4N2M0), MET-positive, PD-L1-positive (TPS50%), with ipsilateral mediastinal lymph node involvement; and Right Renal Mass: Suspicious for Clear Cell Renal Carcinoma.

**Figure 4.**
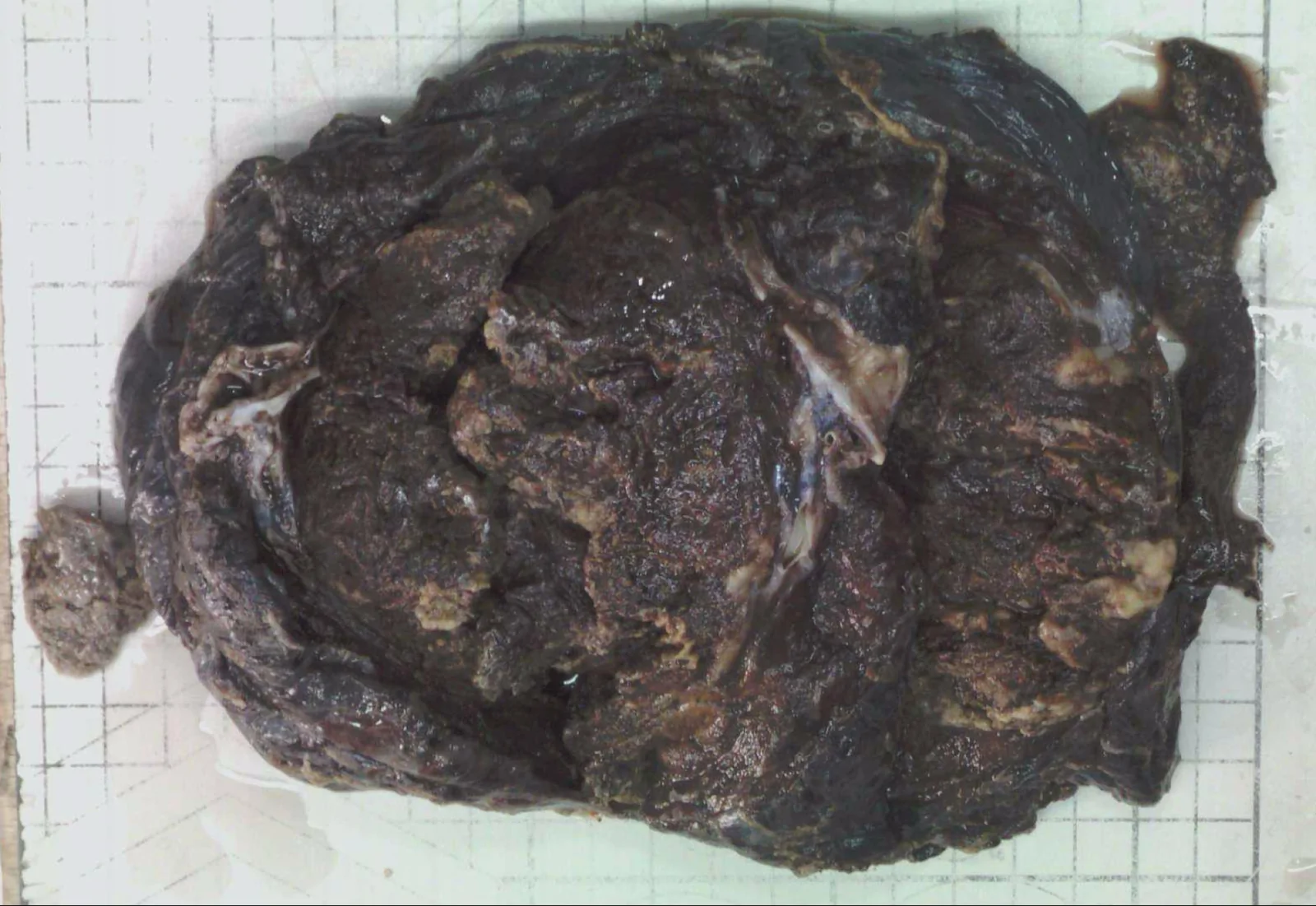
Gross pathological specimen of the resected right upper lobe tumor. The specimen appears as a grayish-red mass with extensive necrotic tissue, cystic degeneration, and a spongy appearance. The cut surface has a paste-like consistency and is friable. (original size).

**Figure 5.**
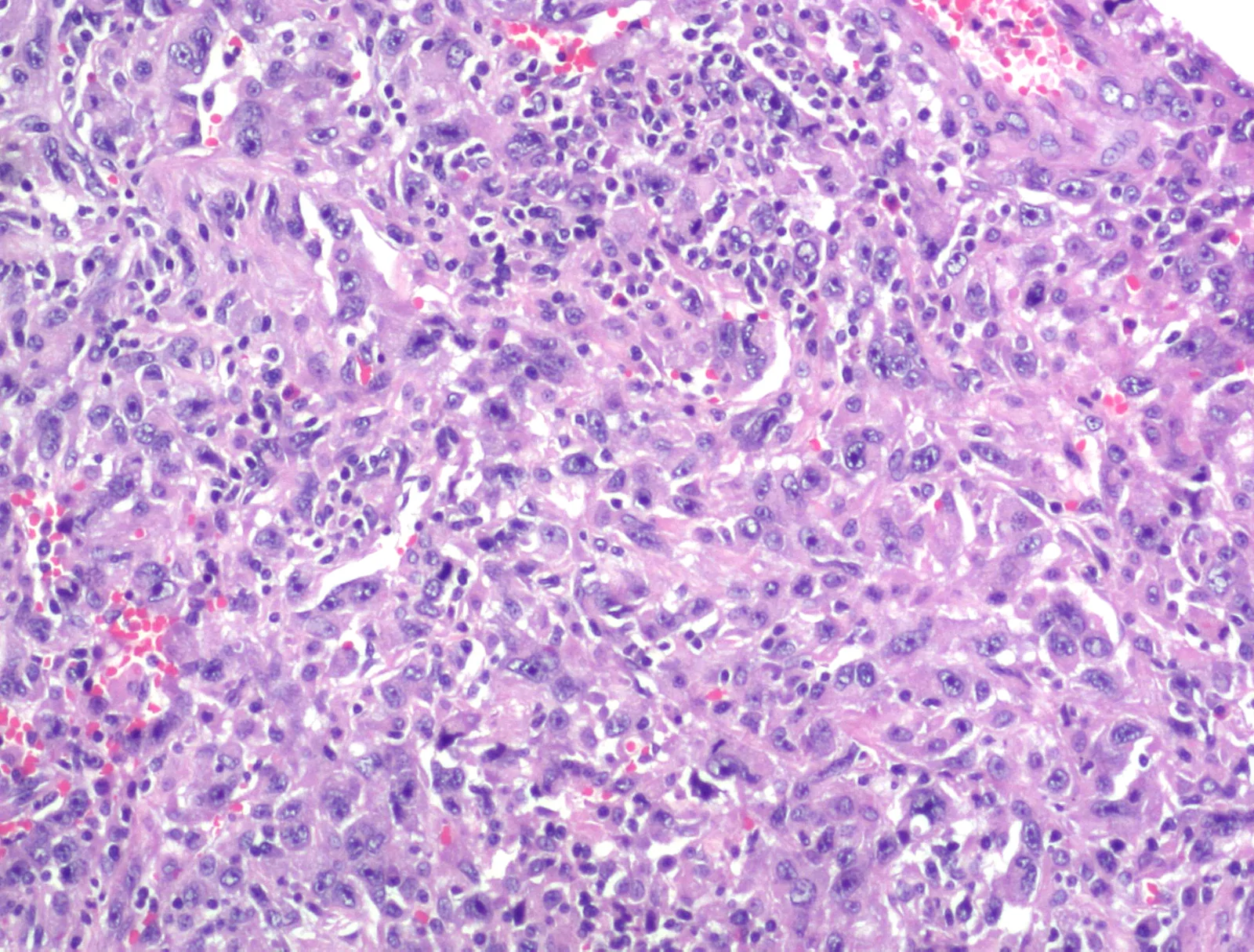
Histopathological examination of the resected specimen (hematoxylin and eosin stain) reveals extensive geographic tumor necrosis, perineural and lymphovascular invasion, and tumor spread through air spaces (STAS). All surgical margins, including the bronchial, vascular, and staple line, are free of carcinoma (original magnification, ×200). STAS = spread through air spaces.

On September 20, 2025, the patient returned for a follow-up examination approximately 1 month postoperatively. Physical examination revealed a well-healed, 10-cm surgical scar on the right chest wall. A soft, poorly mobile mass, measuring approximately 2 cm × 3 cm, was palpable at the mid-portion of the scar. Breath sounds were coarse bilaterally without audible rales or rhonchi. Laboratory tests showed a leukocyte count of 10.90 × 10^9^/L, hemoglobin of 102 g/L, platelet count of 399 × 10^9^/L, neutrophil count of 8.69 × 10^9^/L, elevated CRP (91.85 mg/L), and hypoalbuminemia (32.9 g/L). Tumor markers, renal function, and coagulation profiles were within normal limits. Ultrasonography identified right supraclavicular lymphadenopathy. Combined thoracic and abdominal CT scans revealed: status post right upper lobectomy with new, significantly enlarged mediastinal lymph nodes causing tracheal compression and stenosis (Fig. 1E, F); and bilateral adrenal metastases, the largest measuring approximately 5.1 cm × 4.2 cm. A whole-body bone scan showed no evidence of osseous metastasis. Consultation with the thoracic surgery team concluded that the palpable chest wall mass was likely an inflammatory reaction to surgical sutures, requiring no specific intervention. The patient’s significant dyspnea was attributed to tracheal compression and stenosis. Bronchoscopic stent placement was recommended to maintain airway patency; however, the patient declined both this procedure and initiation of targeted therapy due to financial constraints. After comprehensive evaluation, systemic anti-tumor therapy was initiated on September 25, 2025. The regimen consisted of toripalimab (240 mg IV), docetaxel (100 mg IV), and lobaplatin (45 mg IV), administered in 21-day cycles. The first cycle was completed without complications. Following the initial cycle of combined immunotherapy and chemotherapy, treatment was adjusted in early October 2025 to include boretinib, a MET inhibitor, administered orally at a dose of 200 mg twice daily. A follow-up CT scan of the chest and abdomen on November 1, 2025, demonstrated a favorable response: reduction in the size of the metastatic mediastinal lymph nodes with consequent alleviation of tracheal compression; and a decrease in the size of the bilateral adrenal metastases. The previously noted right renal masses remained stable. Based on this response, the patient received 2 additional cycles of combination therapy on November 3 and November 24, 2025, consisting of boretinib, docetaxel (100 mg IV), and lobaplatin (45 mg IV). On December 6, 2025, laboratory tests during an outpatient visit revealed severe myelosuppression, with a leukocyte count of 0.49 × 10^9^/L. Hemoglobin was 115 g/L. The patient received supportive therapy with granulocyte colony-stimulating factor (G-CSF). By the follow-up assessment on December 26, 2025, the leukocyte count had recovered to 4.46 × 10^9^/L. Repeat CT imaging showed continued improvement, with further shrinkage of both the mediastinal lymphadenopathy and the adrenal metastases (Fig. 1G–I). In response to the episode of severe myelosuppression, the subsequent chemotherapy regimen was modified. Beginning with the cycle on December 26, 2025, treatment consisted of boretinib combined with docetaxel (90 mg IV) and carboplatin (380 mg IV, dosed by target AUC). This adjusted regimen was continued, with the sixth cycle administered on January 18, 2026. At the most recent assessment, the patient has maintained a partial response (PR) for over 6 months. He is asymptomatic, reporting no cough, dyspnea, or sputum production. His general condition is good, with satisfactory energy levels, appetite, and sleep. The patient’s entire disease course is as outlined in Timeline (Table S1, Supplemental Digital Content 1).

## 3. Discussion

The coexistence of a METex14 mutation and high PD‑L1 expression (≥60%) in this case of PSC – the most aggressive non‑small cell lung cancer subtype – raises important questions about optimal adjuvant strategy. While both biomarkers individually predict response to targeted therapy or immunotherapy, their concurrent presence and the optimal timing of postoperative systemic treatment in PSC remain underexplored. The present case is notable not only for this dual positivity but also for its exceptionally rapid postoperative progression, including mediastinal lymphadenopathy causing tracheal compression and bilateral adrenal metastases within 1 month after resection. This fulminant course aligns with that reported by Jiyun Lee et al,^[2]^ where a patient who declined adjuvant therapy developed a 16 cm thoracic recurrence within 2 months, suggesting that PSC may have a narrower therapeutic window than other NSCLC subtypes. Current Chinese guidelines for NSCLC^[3]^ recommend that patients with actionable driver mutations or high PD‑L1 expression who are in satisfactory condition may receive adjuvant targeted therapy within 4 to 8 weeks postoperatively. However, the accelerated relapse pattern observed in our case and in the literature implies that for PSC, especially with dual biomarkers, earlier initiation of systemic therapy – potentially before the conventional postoperative window – might be critical to delay progression and improve tumor control, a hypothesis that warrants further investigation in larger cohorts.

The clinical presentation of PSC is nonspecific, and tumor markers are often not elevated. Chest CT typically reveals a large mass, but definitive diagnosis relies on histopathological examination. Notably, the WHO Classification of Thoracic Tumors explicitly states that PSC is defined based on surgical resection specimens,^[4]^ as cytology and small biopsy samples frequently lead to misdiagnosis or underdiagnosis. Although definitive diagnosis ideally depends on surgical specimens and comprehensive immunohistochemistry, in clinical practice, most advanced PSC cases are diagnosed using limited biopsy material. In the present case, the initial bronchoscopic biopsy suggested inflammatory changes, representing likely a false-negative result. A subsequent CT-guided biopsy revealed scattered, highly atypical tumor cells suggestive of, but not diagnostic for, sarcomatoid carcinoma. A definitive diagnosis of pleomorphic carcinoma was only established postoperatively following multidisciplinary discussion and surgical resection. However, the postoperative course was marked by rapid recurrence, manifesting as lymph node enlargement causing tracheal compression and adrenal metastases. This outcome may be attributed to factors including surgical technique, completeness of resection and lymphadenectomy, and the intrinsically aggressive biology of PSC. From a therapeutic standpoint, the surgical intervention in this case failed to achieve durable local control. This underscores that for stage IIIB PSC where complete resection with lymph node dissection is not feasible, surgery is likely contraindicated. Nevertheless, for patients with an unclassifiable tumor on small biopsy, surgical resection can serve a dual purpose of establishing a definitive diagnosis and debulking the tumor, though this approach must be considered with extreme caution, particularly when PSC is suspected. Whether repeated or more extensive biopsies could improve the preoperative diagnostic yield for PSC warrants further investigation. Furthermore, genomic profiling in this case identified a MET exon 14 skipping mutation and high PD-L1 expression. Existing literature reports that actionable genetic alterations occur in up to 45% of PSC cases,^[5]^ and PD-L1 expression is more prevalent in PSC than in conventional NSCLC subtypes.^[6]^ Therefore, aggressive pursuit of comprehensive molecular profiling and PD-L1 testing is particularly crucial for this aggressive tumor type.

The primary treatment for early-stage PSC is surgical resection, as chemotherapy and radiotherapy are associated with limited efficacy, and neither adjuvant chemotherapy nor radiotherapy has been shown to provide a significant survival benefit.^[7]^ In recent years, some progress has been made in immunotherapy and targeted therapy for PSC. However, the optimal treatment strategy for PSC patients with concurrent MET mutations and high PD-L1 expression remains undefined. There is currently insufficient data to determine whether immune checkpoint inhibitor(s) (ICIs) are effective after progression on targeted therapy, whether ICIs should be used first, followed by targeted therapy upon progression, or whether combined ICIs and targeted therapy could yield improved survival benefits. Studies have reported that in lung adenocarcinoma patients with both MET mutations and high PD-L1 expression, concurrent use of targeted therapy and immunotherapy led to rapid symptomatic improvement and longer progression-free survival (PFS), but with a notable increase in immune-related adverse events. PSC is believed to arise through an epithelial-mesenchymal transition (EMT) process, in which epithelial-like cells transform into sarcomatoid cells. While MET mutations are observed in lung adenocarcinoma, their incidence is higher in PSC.^[8]^ MET proto-oncogene alterations include METex14, gene amplification, and overexpression. Among these, METex14 skipping mutations are the most common and well-studied variant and are considered a distinctive molecular feature of PSC. It is estimated that approximately 90% of PSC patients with METex14 mutations develop distant metastases.^[9]^ A clinical study investigating savolitinib in METex14-mutated lung cancer included 25 PSC patients, revealing an objective response rate of 40% and a median PFS of 5.5 months in this subgroup.^[4]^ In contrast, the study by Tsuda et al suggested that targeted therapy may confer a longer overall survival in PSC (16.2 months vs 17.9 months).^[10]^ However, a large multicenter study reported that for PSC patients with concurrent MET exon 14 skipping mutations and PD-L1 expression, outcomes with immune checkpoint inhibitors (ICI) appeared relatively superior to those with targeted therapy.^[11]^ Although the overall study was multicenter, the subgroup relevant to this comparison comprised only 10 patients; therefore, this exploratory finding should be interpreted with caution. Research by Mayenga et al indicated that patients with METex14-mutated, PD-L1-positive non-small cell lung cancer could still benefit from ICIs following acquired resistance to targeted therapy.^[12]^ Furthermore, the tumor immune microenvironment in PSC is characterized by significant immune cell infiltration and PD-L1 expression, leading to a high prevalence of PD-L1 positivity or high expression in advanced disease,^[13]^ which is generally associated with better responses to ICIs. Reports indicate that the PD-L1 positivity rate in PSC patients is not <45%.^[5]^ Concurrently, vascular invasion is a prominent feature of PSC.^[14]^ Several studies have demonstrated favorable outcomes with immunotherapy‑based regimens – including maintenance, chemo‑immunotherapy, and anti‑angiogenic combinations – in PSC, suggesting that the addition of anti‑angiogenic agents may offer further clinical benefit.^[15-18]^ In summary, for PSC patients with concurrent METex14 mutations and high PD-L1 expression, prognosis appears more favorable compared to other PSC subtypes. However, whether targeted therapy or immunotherapy should be preferred first-line therapy remains controversial. While combining targeted and immunotherapeutic agents may lead to more rapid symptom relief, it is also associated with a significantly higher incidence of adverse events. Therefore, additional randomized controlled trials are warranted to explore optimal treatment strategies. In the present case, guided by the 2025 CSCO guidelines for NSCLC, the patient received postoperative adjuvant therapy with boretinib (a MET inhibitor) combined with chemotherapy. Immunotherapy is planned for consideration upon development of resistance to targeted therapy.

## 4. Conclusion

This case underscores the aggressive nature of stage IIIB PSC and serves as a cautionary illustration that surgical resection alone may be insufficient, given the rapid postoperative recurrence observed despite complete removal. A more cautious approach to surgical indication in this specific setting may be warranted. Comprehensive genomic sequencing and PD‑L1 testing are essential for identifying actionable targets, as the high frequency of alterations in PSC expands therapeutic possibilities. While concurrent METex14 mutation and high PD‑L1 expression may predict favorable treatment responsiveness, the optimal first‑line strategy – prioritizing targeted therapy or immunotherapy – remains unclear. Combination approaches warrant further investigation, though this single case cannot guide that decision, and larger studies are needed.

## Acknowledgments

We sincerely acknowledge the Department of Radiology of the People’s Liberation Army Joint Logistics Support Force No. 924 Hospital for providing imaging data, valuable pathological data from the Department of Pathology, and the patients who participated in this study.

## Author contributions

**Conceptualization:** Zhen Xiong.

**Data curation:** Zhen Xiong.

**Project administration:** Zhen Xiong.

**Writing – original draft:** Junzhang Qin.

**Writing – review & editing:** Junzhang Qin.


